# Is Screening of TORCH Worthwhile in Women with Bad Obstetric History: An Observation from Eastern Nepal

**DOI:** 10.3329/jhpn.v29i1.7569

**Published:** 2011-02

**Authors:** Namrata Kumari, Norman Morris, Renu Dutta

**Affiliations:** ^1^ Department of Microbiology, Indira Gandhi Institute of Medical Sciences, Patna, Bihar, India; ^2^ Department of Obstetrics and Gynaecology, B.P. Koirala Institute of Health Sciences, Dharan, Nepal; ^3^ Department of Microbiology, B.P. Koirala Institute of Health Sciences, Dharan, Nepal

**Keywords:** Bad obstetric history, Case-control studies, TORCH screening, Nepal

## Abstract

This pilot case-control study at a tertiary-care hospital over a four-month period was aimed at evaluating the possible usefulness of screening of TORCH (*Toxoplasma gondii*, rubella virus, cytomegalovirus, and Herpes simplex virus) in females with bad obstetric history. The study included 12 women with bad obstetric history and a similar number of matched controls with previous normal pregnancies. A serological evaluation of TORCH infections was carried out by detecting IgG and IgM antibodies against these infections by ELISA test-kit. Statistical analysis was not done to compare the results relating to the two groups due to a small number of cases and controls included in the study. Ten (83.3%) of the 12 cases with bad obstetric history and two (16.7%) of the 12 healthy controls were serologically positive at least for one of the TORCH agents. The seropositivity rate in women with bad obstetric history was quite high compared to that in the normal healthy controls. The results suggest that a previous history of pregnancy wastage and the serological evaluation of TORCH infections during current pregnancy must be considered while managing cases with bad obstetric history.

## INTRODUCTION

Bad obstetric history (BOH) implies previous unfavourable foetal outcome in terms of two or more consecutive spontaneous abortions, history of intrauterine foetal death, intrauterine growth retardation, stillbirth, early neonatal death and/or congenital anomalies. Cause of BOH may be genetic, hormonal, abnormal maternal immune response, and maternal infection (1). Primary infections caused by TORCH—*Toxoplasma gondii*, rubella virus, cytomegalovirus (CMV), and Herpes simplex virus (HSV)—is the major cause of BOH (2).

The prevalence of these infections varies from one geographical area to another (3). These maternalinfections are initially unapparent or asymptomatic and are, thus, difficult to diagnose on clinical grounds (4, 5). Therefore, diagnosis of acute TORCH infection in pregnant women is usually established by demonstration of seroconversion in paired sera or by demonstration of specific IgM antibodies (1). Enzyme-linked immunosorbent assay (ELISA) for IgM antibodies against these infections is highly sensitive and specific (6). The conventional single serum assays do not make a clear distinction between a recent primary and chronic infection. The tendency of specific IgM to persist for a long time even at high levels has been verified in several studies (7, 8). After its introduction in serodiagnosis of *Toxoplasma-*associated infections, the measurement of IgG avidity has proved to be a highly-useful procedure, especially in combination with conventional serological assays (9).

However, this pilot study was aimed at evaluating the possible usefulness of conventional screening of TORCH in females with BOH in eastern Nepal.

## MATERIALS AND METHODS

### Study setting and design

This pilot case-control study was carried out over a four-month period at a tertiary referral hospital in Nepal.

Twelve women with BOH and a similar number of matched controls (without any BOH and previous normal pregnancies) attending our antenatal clinic were included in the study. Cases were selecteddepending on a previous history of having 2-3 pregnancy wastages, intrauterine deaths, preterm deliveries, intrauterine growth retardation, and unexplained early neonatal deaths.

Factors considered for selecting matched controls were age (±2 years), similar gravidity, same period of gestation in present pregnancy, and same number of livebirths opposed to bad obstetric complaints in the cases group. Detailed examinations and conventional laboratory investigations were carried out in both the groups. After taking consent from each woman, 3 mL of venous blood was collected in a container with strict aseptic precautions. The serum, thus, obtained was used for serological evaluation of TORCH infections. TORCH IgG and IgM antibodies were detected from the serum by ELISA test-kit (Calbiotech Inc, Canada) following the instructions of the manufacturer. The kits for detection of antibodies were based on sandwich ELISA method. Absorbance was taken at wavelength of 450 nm, and results were calculated according to the instructions. TORCH antibody index was calculated by dividing the value of each sample by calibrator values. The antibody index of 1.0 or greater was considered positive for antibodies.

The results of the two groups (cases and controls) were compared (Table 1). Statistical analysis was not done to compare the results relating to the two groups due to a small number of cases and controls included in the study.

**Table 1.** Different presentations of BOH cases and matched controls with TORCH status

Case vs control	Age (years)	Parity	Live-birth(s)	Abort-ion(s)	Still-birth(s)	Neonatal death(s)	TORCHstatus
Case	24	1	0	1	0	1	All negative
Control	26	2	2	0	0	0	*T.gondii* positive
Case	22	1	0	1	1	0	Rubella virus positive
Control	21	2	2	0	0	0	All negative
Case	29	2	0	0	1	1	*T. gondii*, CMV, and HSV positive
Control	28	2	2	0	0	0	All negative
Case	23	2	0	0	0	2	Rubella virus positive
Control	25	2	2	0	0	0	All negative
Control	24	3	3	0	0	0	All negative
Case	22	2	0	1	0	2	*T. gondii* and rubella virus positive
Case	20	1	0	2	0	1	Rubella virus and HSV positive
Control	21	3	3	0	0	0	All negative
Case	18	0	0	2	0	0	*T. gondii* and rubella virus positive, HSVpositive
Control	20	2	2	0	0	0	All negative
Case	24	1	0	1	1	0	*T. gondii* positive
Control	25	2	2	0	0	0	All negative
Case	30	3	0	0	0	3	Rubella viruspositive
Control	31	3	3	0	0	0	All negative
Case	35	0	0	2	0	0	All negative
Control	37	2	2	0	0	0	*T. gondii* positive
Case	18	0	0	2	0	0	*T. gondii* positive and HSV positive
Control	20	2	2	0	0	0	All negative
Case	22	0	0	2	0	0	*T. gondii* positive
Control	23	2	2	0	0	0	All negative

All=*T. gondii*, rubella, and HSV; BOH=Bad obstetric history; CMV=Cytomegalovirus; HSV=Herpes simplex virus 2; *T. gondii*=*Toxoplasma gondii*; TORCH=*Toxoplasma gondii*, rubella virus, cytomegalovirus, and Herpes simplex virus

### Ethical approval

The Ethical Committee of the Institute granted ethical clearance to the study.

## RESULTS

Different presentations of BOH cases and matched controls with their TORCH status are shown in Table 1, and seropositivity of TORCH agents in the two groups is shown in Table 2. Five cases showed mixed seropositivity. Mixed seropositivity was not found in any of the controls. A maximum number of BOH cases was found in females aged 18-24 years.

**Table 2.** Seropositivity of TORCH agents in the two groups

TORCH agent	Seropositivity in BOH group (n=12)		Seropositivity in control group (n=12)	
No.	%	No.	%
*Toxoplasma gondii*	6	50	2	16.7
Rubella virus	6	50	0	0
Cytomegalovirus	1	8.3	0	0
Herpes simplex virus 2	4	33.3	0	0

BOH=Bad obstetric history; TORCH=*Toxoplasma gondii*, rubella virus, cytomegalovirus, and Herpessimplex virus

Ten (83.3%) of the 12 BOH cases and two (16.7%) of the 12 healthy controls were serologically positive at least for one of the TORCH agents. The seropositivity rate in women with BOH was quite high compared to that in the normal healthy controls.

In the BOH cases, the seropositivity for *T. gondii* was 50%, rubella virus 50%, HSV-2 33.3%, and CMV 8.3% whereas, in the control cases, the seropositivity for *T. gondii* was 16.7% only and that of the remaining ones was 0%.

## DISCUSSION

To the best of our knowledge, baseline data on seropositivity in the local population are not available from any part of Nepal. In the present study, *T. gondii* (50%), rubella virus (50%), HSV-2 (33.3%), and CMV (8.3%) were found in pregnant women with BOH. These data are very high compared to those available from the neighbouring country (India) (3, 7). It might be due to the fact that the number of cases and controls included in the present study is very less, and, as such, it may not be reflect the true picture of TORCH infections. However, the difference between the two groups was quite significant numerically.

Although IgG of the samples was determined, the interpretation is mainly based on the value of IgM as paired sera were not tested.

Further, the cost of the whole TORCH-panel test being very high, most general population of an underdeveloped country, such as Nepal, cannot comfortably afford. By including vaccination against rubella virus (MMR vaccine) in the national immunization schedule, the incidence of congenital rubella can be reduced to a large extent. Moreover, this component can be deleted from the TORCH-panel investigations, thereby reducing their cost.

### Conclusions

Based on the findings of the study, it is concluded that a previous history of pregnancy wastage and the serological reactions for TORCH infections during current pregnancy must be considered while managing BOH cases to reduce the adverse foetal outcome. Keeping consideration of the high cost of the test panel, selected tests (of the whole panel) are recommended on an individual case basis. Incorporation of rubella immunization into the national immunization schedule is recommended. *Toxoplasma*-associated infection can be prevented by educating the public about avoidance of ingestion of raw or insufficiently-cooked meat and poultry and keeping proper hygiene. An extensive study covering a large population should be conducted to know the seropositivity of TORCH agents and also to know the real status of these infections in BOH cases.
